# *Armillaria luteo-virens Sacc* Ameliorates Dextran Sulfate Sodium Induced Colitis through Modulation of Gut Microbiota and Microbiota-Related Bile Acids

**DOI:** 10.3390/nu13113926

**Published:** 2021-11-03

**Authors:** Nana Zhang, Jianlin Liu, Xinxin Guo, Shuying Li, Fengzhong Wang, Minjie Wang

**Affiliations:** 1 Institute of Food Science and Technology, Chinese Academy of Agricultural Sciences, Ministry of Agriculture, Beijing 100090, China; zhangnn16@163.com (N.Z.); queen_jianlin@163.com (J.L.); guoxx26@163.com (X.G.); 2 Key Laboratory of Agro-Products Processing, Chinese Academy of Agricultural Sciences, Beijing 100090, China; 3 School of Basic Medical Sciences, Inner Mongolia Medical University, Huhehaote 010107, China; Wangmingjienmg@163.com

**Keywords:** *Armillaria luteo-virens Sacc*, colitis, gut microbes, bile acid, intestinal barrier, inflammation

## Abstract

*Armillaria luteo-virens Sacc* (ALS) is a rare wild Chinese medicinal and edible basidiomycete. However, its protective effect on intestinal functions and the underlying mechanism is still unknown. This work explored the improvement of dextran sulfate sodium (DSS)-induced colitis by ALS. ALS supplementation markedly improved colitis symptoms, gut barrier integrity, and goblet loss in DSS-treated mice. In addition, ALS inhibited colonic inflammation through the inhibition/activation of the mitogen-activated protein kinases/NF-κB signaling pathway. The 16S rRNA gene-based microbiota analysis revealed that ALS altered the gut microbiota composition, decreasing the richness of *Enterobacteriaceae* and increasing the abundance of *Lactobacillaceae*. The bile-acid-targeted metabolomic analysis showed that ALS recovered the microbial bile acid metabolism in the gut, enabling the activation of the farnesoid X receptor signaling by these acids, thus maintaining the intestinal homeostasis. Importantly, broad-spectrum antibiotic treatment reduced the efficacy of ALS-induced protection from colitis. Overall, our findings suggest that ALS may represent a novel approach in the nutritional intervention to prevent colitis.

## 1. Introduction

Inflammatory bowel disease (IBD), including ulcerative colitis and Crohn’s, is a chronic and relapsing inflammatory disease of the intestine [1]. Discontinuous lesions are the major pathologic manifestation of ulcerative colitis [2]. The prevalence of IBD is increasing not only in developed countries but also in developing countries and has emerged as a global public health issue [3]. Recent studies indicate that IBD pathogenesis is closely related to intestinal barrier disruption, gut microbiome imbalance, and subsequent mucosal dysregulated responses to the intestinal microbiota, although the etiology and pathogenesis of IBD are still unclear [4,5].

The treatment of clinical IBD symptoms through the inhibition of the immune response is the main approach in traditional IBD interventions. Most of the available therapeutic options cause off-target side effects [6]. The underlying etiological factors are generally not addressed, such as the mucus layer loss, intestinal barrier disruption, and gut microbiota dysbiosis in the intestinal tract. A high probability of recurrence remains present during clinical treatments [7]. Therefore, the development of additional therapeutic or preventive approaches is urgent in order to prevent and treat colitis.

The gut microbiota, containing trillions of microbes, can be shaped by diet and is a crucial environmental factor affecting host physiology [8]. The gut microbiome composition and related metabolites are significantly transformed in murine colitis models as well as IBD patients, including *Enterobacteriaceae* enrichment and Lactobacillus decrease [9]. Previous findings showed that microbiota transplantation from IBD patients to germ-free mice alters the mouse intestinal barrier functions [10]. Supplementation of *Lactobacillus* significantly reduces inflammatory responses in a murine colitis mode [11]. Focusing on the modulation of gut microbiota may be promising for preventing and treating colitis.

*Armillaria luteo-virens Sacc* (known as *Floccularia luteovirens*) is a rare Chinese medicinal and edible basidiomycete endemic in the Qinghai–Tibet plateau and is harvested only once a year in the wild [12]. *Armillaria luteo-virens Sacc* (ALS) is an edible fungus well known for its unique taste, flavor, and diverse nutrients [13]. Proteins, amino acids, and microelements are abundant in ALS. As a component of traditional Tibetan medicine prescriptions, it is also used to treat neurological diseases such as neurasthenia, dizziness, insomnia, headaches, infantile convulsions, and numbness of the limbs [14]. Evidence from in vitro studies indicated that ALS extracts have anti-tumor and antioxidant properties [12,15]. However, few studies, if any, have documented the use of ALS in gastrointestinal disorders. Therefore, the present research investigated the impact of ALS administration on the amelioration of colitis through the establishment of a DSS-induced colitis model in mice. The role of the gut microbiota in modulating the effects of ALS on host intestinal functions was also explored.

## 2. Materials and Methods

### 2.1. Preparation of ALS Powder

The fruiting bodies of ALS used in this study were provided by the Tibet Academy of Agricultural and Animal Husbandry Sciences. The fresh ALS bodies were washed, cut into pieces, freeze-dried by the OE-950 (Labor, Budapest, Hungary), and ground into powder. The proximate profile of the ALS powder is available online in Table S1.

### 2.2. Mice and Dietary Supplementation

The animal experiment was approved by the Animal Care and Use Committee of the School of Basic Medical Sciences, Inner Mongolia Medical University. The animal care and handling were performed following the guidelines of the national standards outlined in the “Laboratory Animal Requirements of Environment and Housing Facilities” (GB 14925-2010). Eight-week-old male Crl: CD1(ICR) mice were obtained from the Vital River Laboratory Animal Technology (Beijing, China) and housed under specific pathogen-free (SPF) conditions: 25 ± 1 °C, 55 ± 5% humidity, and 12 h light/dark cycles. After 1 week of acclimation, mice were randomly divided into three experimental groups (ten mice per group) and fed with a standard chow diet or an isocaloric diet for 45 days, in which ALS freeze-dried powder was supplemented at a ratio of 8% (Table S2). The experimental groups are as follows: (1) Control group: Fed daily with a standard chow diet; (2) DSS group: 2% DSS in drinking water for the last 5 days; (3) ALS + DSS group: Fed daily with an 8%ALS diet starting 40 days before colitis induction and maintained during colitis induction. Regarding the antibiotic treatment, broad-spectrum antibiotics (ampicillin 1 g/L, vancomycin 0.25 g/L, neomycin 1 g/L, and metronidazole 1 g/L) were added to the drinking water, which was renewed every 3 days and maintained with the same composition until the end of the experiment [16].

### 2.3. Induction of Colitis

**Figure 1:**
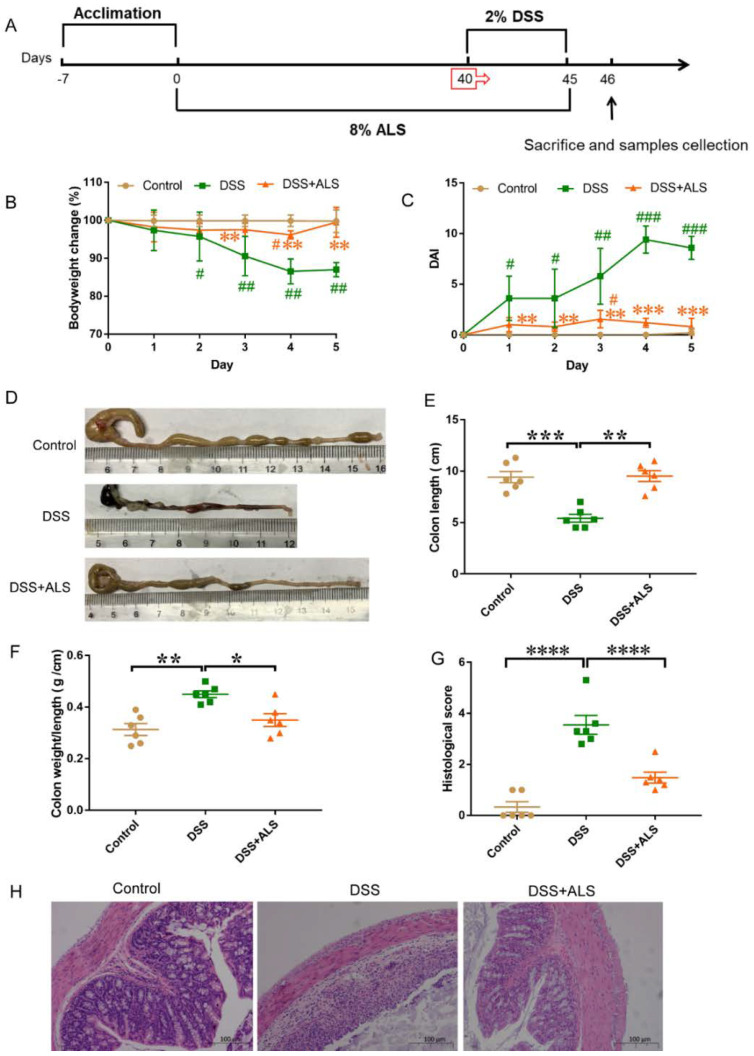
ALS alleviates colitis symptoms. (**A**) Study design of the experiment. (**B**) Body weight change during the induction of colitis. Data are mean ± SEM. *n* = 6 per group. # *p* < 0.05, ## *p* < 0.01, relative to control group; ** *p* < 0.01, relative to dextran sodium sulfate (DSS) group. (**C**) DAI scores during DSS-induced period. Data are mean ± SEM. *n* = 6 per group. Data are mean ± SEM. *n* = 6 per group. # *p* < 0.05, ## *p* < 0.01, ### *p* < 0.001 relative to control group; ** *p* < 0.01, *** *p* < 0.001, relative to DSS group. (**D**) Pictures of colon samples showing the colon length. (**E**) Colon length. (**F**) Colon weight/length ratio. (**G**) Histological scores of the H&E-stained sections. (**H**) H&E-stained colonic sections. Scale bar = 100 μm. Data are mean ± SEM. *n* = 6 per group. * *p* < 0.05, ** *p* < 0.01, *** *p* < 0.001 and **** *p* < 0.0001.

All mice, except the control group, received 2% dextran sodium sulfate (DSS) (molecular weight 36∓50 kDa, MP Biomedicals, Santa Ana, CA, USA) dissolved in drinking water for 5 days to induce chemical colitis (Figure 1A). The bodyweight of each animal was monitored each day and signs of rectal bleeding and stool consistency were recorded. The colitis disease activity index (DAI) was evaluated through the combination of the parameters of stool consistency, weight loss, and rectal bleeding, as previously described [17]. The colon length was measured after the mice were sacrificed.

### 2.4. Quantitative Real-Time PCR

Total RNA was extracted from the colonic tissue using Trizol (Invitrogen, Carlsbad, CA, USA). cDNA was obtained using the GoScript reverse transcription system (Promega, Madison, WI, USA). A real-time PCR system (ABI 7500) was used to perform qPCR. The sequences of the primers used in this study are listed in Table S3. The relative expression of the target genes was calculated using GAPDH as the reference gene.

### 2.5. Western Blotting

Colon tissues were lysed using a radioimmunoprecipitation assay (RIPA). The protein concentration was detected using the BCA protein assay kit (Life Technologies, Eugene, OR, USA). Thirty milligrams of the proteins were separated by electrophoresis on a 10% SDS polyacrylamide gel and then transferred to PVDF membranes. The samples were incubated with primary antibodies against phospho-ERK1/2 (Cell Signaling Technology, Boston, MA, USA), anti-total ERK1/2 (Cell Signaling Technology), phospho-JNK (Cell Signaling Technology), anti-total JNK (Cell Signaling Technology), phospho-p38 (Cell Signaling Technology), anti-total p38 (Cell Signaling Technology), phospho-NF-κB (Cell Signaling Technology), anti-total NF-κB (Cell Signaling Technology), and HSP90 (Cell Signaling Technology). Proteins were detected using the HRP-conjugated secondary antibody and the chemiluminescent HRP substrate (Millipore, Billerica, MA, USA).

### 2.6. Immunofluorescent Staining

Colon tissues were permeabilized using Tris-buffered saline (TBS) containing 0.3% Triton X-100 and incubated at room temperature for 30 min. The tissues were then boiled in Tris EDTA (pH 9.0) for 20 min, blocked with 5% bovine serum albumin in TBS (pH 7.4) for 1 h, then incubated overnight with the primary antibody ZO-1 (Abcam, Cambridge, MA, USA), Occludin (Santa Cruz, Dallas, TX, USA), and Muc2 (Santa Cruz, Dallas, TX, USA) and incubated at 4 °C before the AlexaFluor-488 secondary antibody (Invitrogen, Carlsbad, CA, USA) was used. 

### 2.7. Hematoxylin and Eosin (H&E Staining)

Colon tissues were fixed in 4% paraformaldehyde and embedded in paraffin. The paraffin sections were cut into 4 μm-thick sections that were stained with H&E and observed using an optical microscope. The histopathologic score was obtained according to the following evaluation criteria: Crypt damage (0–4 scale), the severity of inflammation (0–3 scale), and the depth of injury (0–3 scale) [18]. 

### 2.8. Alcian Blue Staining

Fresh colon tissue sections were fixed in 10% buffered formalin. Paraffin was used to embed the tissues and the tissues were stained with Alcian-Blue/Nuclear-Fast-Red for 20 min after dewaxing the tissue section. Goblet cells were viewed under a light microscope (Leica DM500, Chicago, IL, USA). The quantification of goblet cells based on Alcian blue-stained sections was performed using Image J software. Six crypts of each section were randomly selected to evaluate the goblet cell number of crypts.

### 2.9. Transmission Electron Microscopy

Colonic tissues were fixed with 2.5% glutaraldehyde immediately after the mice were sacrificed. The excess fixative was removed using PBS. Osmium tetroxide 1% was used to fix samples at 4 °C for 2 h, and then acetone was used to perform the dehydration. The mixture of Propylene oxide and EPON resin (1:1) was used to infiltrate the samples for 1 h, and then overnight infiltration in 100% EPON’s resin was performed. Lastly, 100% EPON flat molds were used to embed the tissues for 36 h at 60 °C. Samples were observed by an H-7650 transmission electron microscope (Hitachi, Tokyo, Japan).

### 2.10. Analysis of Inflammatory Cytokine and Myeloperoxidase (MPO)

Cytokine levels such as interleukin (IL)-6, tumor-necrosis factor (TNF)-α, and monocyte chemoattractant protein (MCP)-1 in the serum and colon were determined using a ProcartaPlex Mouse Cytokine panel (Thermo Fisher Scientific, Waltham, MA, USA) according to the manufacturer’s instructions and detected using a Luminex MAGPIX System (Luminex, Austin, TX, USA). The colonic MPO activity was assessed using the Mouse MPO Elisa Kit (Abcam, Cambridge, MA, USA) according to the manufacturer’s instructions.

### 2.11. 16S rRNA Gene Sequence Analysis

Total DNA was extracted from the cecum content using a QIAamp-DNA Stool Mini Kit from Qiagen (Hilden, Germany). The ABI GeneAmp^®^9700 PCR System (Applied Biosystems, Foster City, CA, USA) was used to perform PCR amplification. The PCR amplification products were quantified using a QuantiFluorTM-ST Handheld Fluorometer with UV/Blue Channels (Promega Corporation, Madison, WI, USA). The pooled products were quantified and sequenced using the Illumina Miseq platform at Majorbio Bio-Pharm Technology Co., Ltd. (Shanghai, China). The sequences were filtered, trimmed, and classified into operational taxonomic units (OTUs) within 97% similarity before the analysis.

### 2.12. Bile Acid Detection

The targeted bile acid metabolomic analysis from the cecum content was performed using the AB SciexExionLCTMAD HPLC/SciexQTRAP^®^ 6500 MS system (AB Sciex, Framingham, MA, USA). Briefly, 100 mg cecal content was homogenized in liquid nitrogen and dissolved in 900 μL distilled water. The mixture of diluted sample and internal standards was homogenized for 30 s. Next, the samples were centrifuged at 12,000 rpm and 4 °C for 10 min. The residue of the dried supernatant was dissolved in 100 μL water/acetonitrile (*v*/*v* = 8:2) with 0.1% formic acid. The extracted samples were assessed by LC/MS using the multiple-reaction monitoring mode and quantified based on the respective standard curves. 

### 2.13. Statistical Analysis

Statistical analysis was performed using Prism 6.0 (GraphPad Software, La Jolla, CA, USA). Data are presented as the mean ± SEM. Significant differences were evaluated using one-way ANOVA or two-way ANOVA followed by Tukey’s multiple-comparisons test as appropriate. A two-tailed Wilcoxon rank-sum test by R Project was performed to assess the differences of microbiota sequencing data. A value of *p* < 0.05 was considered statistically significant.

## 3. Results

### 3.1. ALS Alleviates Colitis Symptoms

In the present study, the DSS-induced colitis mouse model was used to evaluate the beneficial impacts of ALS on intestinal functions [19]. A standard chow diet or an isocaloric diet in which ALS was supplemented at a ratio of 8% was used to feed the mice, as shown in Figure 1A. DSS treatment significantly reduced bodyweight. The supplementation of ALS exerted a substantial protective effect on bodyweight loss during the colitis induction (Figure 1B). ALS alleviated the reduced disease activity index (DAI) values caused by DSS (Figure 1C). The colon length shortening induced by DSS and the colonic weight/length ratio were significantly improved by the ALS treatment (Figure 1D–F). Consistently, the histological analysis showed that the DSS treatment led to the typical features of colitis: Severe distortion of the crypt structure, infiltrated immune cells, and a loss of goblet cells (Figure 1G,H). ALS treatment protected the colonic epithelium against pathological damage. Together, these results indicated that ALS effectively ameliorated DSS-induced clinical colitis symptoms in a mouse model. 

### 3.2. ALS Improves Intestinal Barrier Function

**Figure 2:**
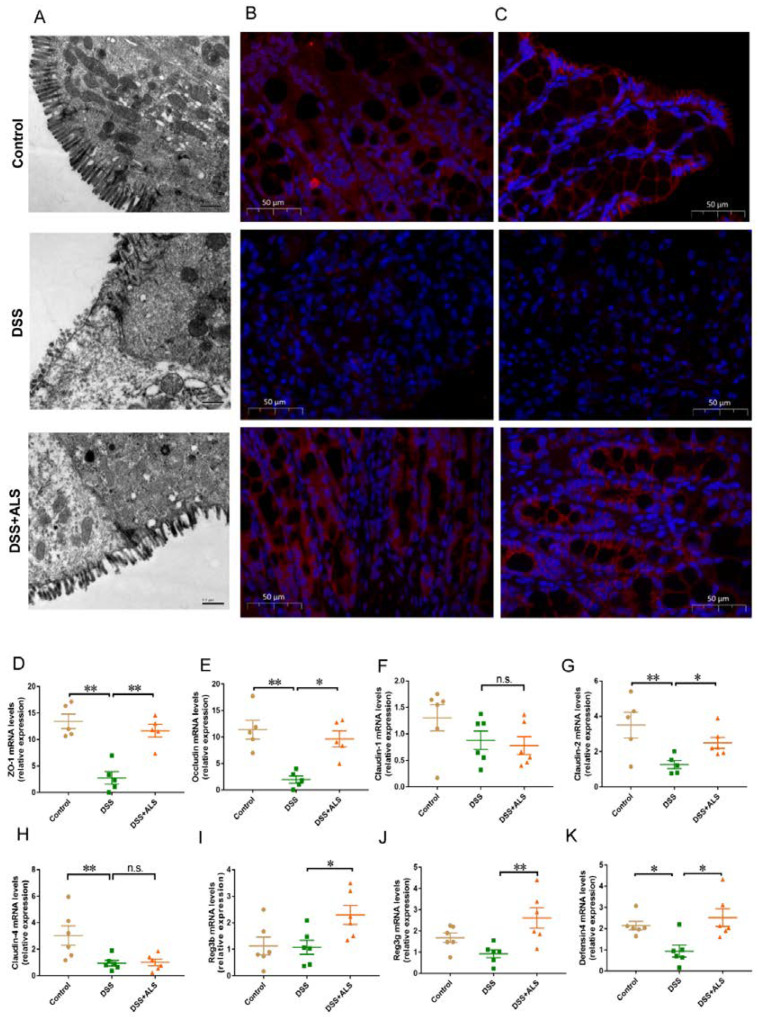
ALS improves intestinal barrier function. (**A**) Representative images of colonic ultrastructure in epithelial cells shown by TEM. (**B**) Representative fluorescent images of ZO-1 in colon. Scale bar = 50 μm. (**C**) Representative fluorescent images of Occludin in colon. Scale bar = 50 μm (**D**) Real-time qPCR analysis of *ZO-1* in colon tissue. (**E**) Real-time qPCR analysis of *Occludin* in colon tissue. (**F**) Real-time qPCR analysis of *claudin-1* in colon tissue. (**G**) Real-time qPCR analysis of *claudin-2* in colon tissue. (**H**) Real-time qPCR analysis of *claudin-4* in colon tissue. (**I**) Real-time qPCR analysis of *Reg3b* in colon tissue. (**J**) Real-time qPCR analysis of *Reg3g* in colon tissue. (**K**) Real-time qPCR analysis of *defensin4* in colon tissue. Data are mean ± SEM. *n* = 5–6 per group. * *p* < 0.05 and ** *p* < 0.01. n.s., not statistically significant.

The first pathological characteristic of colitis was damage of the gut barrier. Then, the impact of ALS on intestinal integrity was studied. A closer examination by transmission electron microscopy showed that DSS-induced colitis mice had more dilated intercellular spaces, disrupted tight right junctions, and shortened microvilli in the colon compared with the mice in the ALS group (Figure 2A). Consistent with the results of TEM, the immunofluorescence analysis in ALS-fed colitis mice revealed an increase in the expression of the tight junction proteins, zonula occludens-1 (ZO-1) (Figure 2B) and occludin (Figure 2C). The mRNA expression of tight junction proteins and antimicrobial peptides was also assessed to evaluate the role of ALS in gut barrier integrity. We found that ALS treatment significantly upregulated the mRNA expression of the tight junction proteins, *ZO-1*, *occludin*, and *Claudin-2* (Figure 2D,E,G). There were no significant differences in the mRNA expression of *Claudin-1* and *Claudin-4* between the ALS + DSS group and the DSS group (Figure 2F,H). The levels of genes encoding for antimicrobial peptides, including regenerating islet-derived 3b (*Reg3b*), *Reg3g*, and *defensin 4* (Figure 2I,J), were increased in ALS-fed mice. These data indicated that ALS effectively improved the structure of the gut barrier disrupted by DSS.

### 3.3. ALS Recovers the Intestinal Goblet Loss

**Figure 3:**
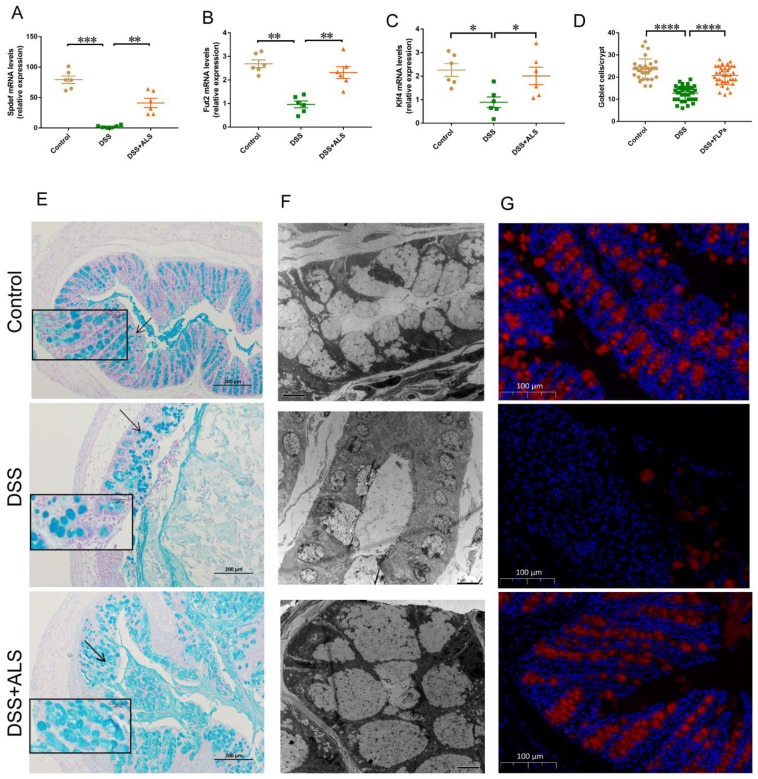
ALS recovers the intestinal goblet loss. (**A**–**C**) The expression of genes related to goblet cell differentiation examined by real-time PCR assay. (**D**) The number of goblet cells was quantified. (**E**) Representative pictures of alcian blue-stained colon. Scale bar = 200 μm. (**F**) Representative TEM images of the mucin granules in colon. Scale bar = 5 μm. (**G**) Representative pictures of immunofluorescence of Muc2 in colon tissues. Scale bar = 100 μm. Data are mean ± SEM. *n* = 5–6 mice per group. * *p* < 0.05, ** *p* < 0.01, *** *p* < 0.001 and **** *p* < 0.0001.

The goblet cells play a decisive role in maintaining the intestinal epithelial layer through the secretion of mucus to provide the first line of defense in response to physical and chemical injury [20,21]. The colons derived from ALS-treated mice displayed a significant increase in key markers of goblet cell differentiation including the SAM pointed domain containing the ETS transcription factor (*Spdef*) promoting the maturation of the goblet, Kruppel-like factor 4 (*Klf4*) regulating goblet cell differentiation, and fucosyltransferase 2 (*Fut2*) encoding fucosyltransferase on the glycosylation of mucins (Figure 3A–C). DSS-colitis mice treated with ALS showed a normalized expression of mucus produced by goblet cells, as shown by Alcian blue staining (Figure 3D,E). Histological examination through transmission electron microscopy revealed the presence of large and entire mucin granules in ALS-fed mice, indicating the accumulation and enhanced biogenesis of the mucin protein in the colonic goblet cells (Figure 3F). Mucin2 (Muc2), the most abundant mucin protein that forms the mucus layer, was heavily glycosylated and was secreted by goblet cells during ALS treatment (Figure 3G). These results suggested that ALS improved intestinal functions by potentially enhancing the mucosal barriers. 

### 3.4. ALS Mitigates Inflammation in DSS-Induced Colitis

**Figure 4:**
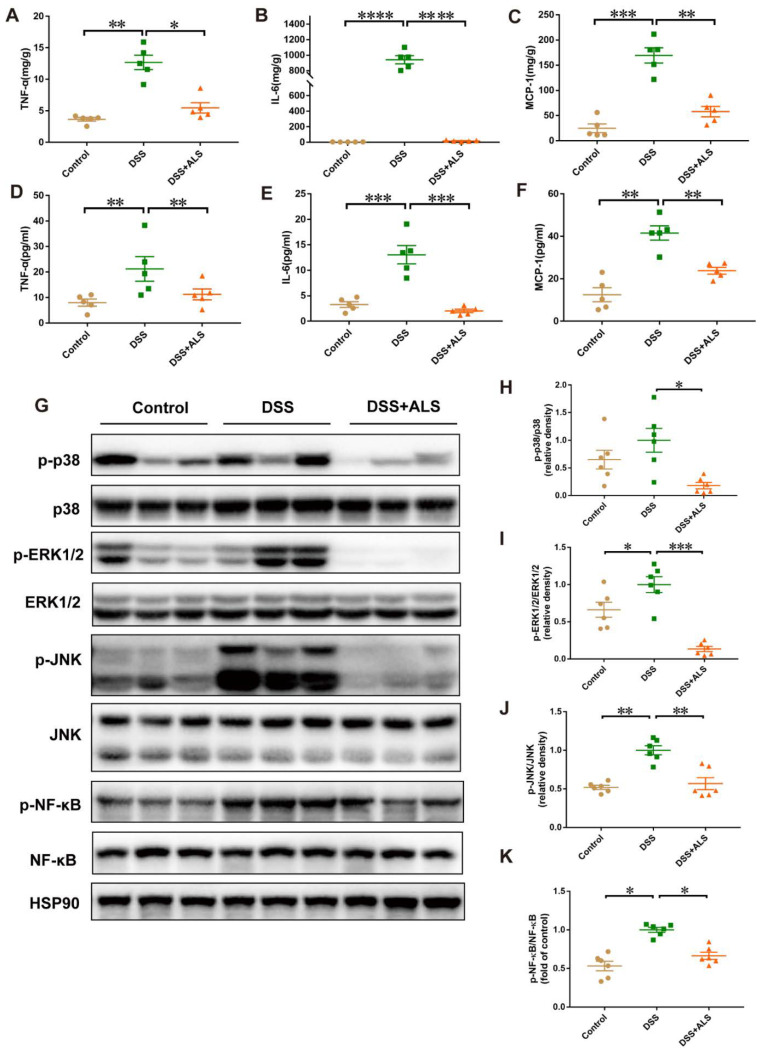
ALS mitigates inflammation in experimental colitis induced by DSS. (**A**–**C**) Concentration of pro-inflammatory cytokines, TNF-α, IL-6, and MCP-1 in colon tissue. (**D**–**F**) Concentration of pro-inflammatory cytokines, TNF-α, IL-6, and MCP-1 in serum. (**G**) Western blot results of p-p38, p38, p-ERK1/2, ERK1/2, p-JNK, JNK, p-NF-κB, and NF-κB. (**H**) Quantification of p-p38/p38 is shown. (**I**) Quantification of p-ERK1/2/ERK1/2 is shown. (**J**) Quantification of p-JNK/JNK is shown. (**K**) Quantification of p-NF-κB /NF-κB is shown. Data are mean ± SEM. *n* = 5–6 per group. * *p* < 0.05, ** *p* < 0.01, *** *p* < 0.001, and **** *p* < 0.0001.

The effect of ALS on inflammatory cytokines was evaluated next and the underlying mechanism was delineated. ALS feeding notably decreased the local levels of pro-inflammatory cytokines, such as TNF-α, IL-6, and MCP-1 in the colonic tissues when compared with the DSS-induced group (Figure 4A–C). A similar change was also observed in serum (Figure 4D–F). Credible studies demonstrated that the mitogen-activated protein kinases (MAPKs) act as important intestinal homeostasis regulators [22]. Consistent with the above effects on the intestinal morphology, the protein levels of phosphorylated extracellular signal-regulated kinase1/2 (p-ERK), phosphorylated p38 (p-p38), and the phosphorylated c-Jun N-terminal kinase (p-JNK) were increased by DSS treatment, and ALS readjusted these changes in our experimental protocol (Figure 4G–J). Since ERK1/2 and JNK regulate the NF-κB pathway, the effect of ALS on NF-κB was further addressed. ALS successfully inhibited the phosphorylation of NF-κB induced by DSS (Figure 4G,K). These findings suggested that ALS exerted a protective effect on DSS-induced colitis through the inhibition of the MAPK/NF-κB signaling pathway.

### 3.5. ALS Ameliorates Gut Microbiota Dysbiosis

**Figure 5:**
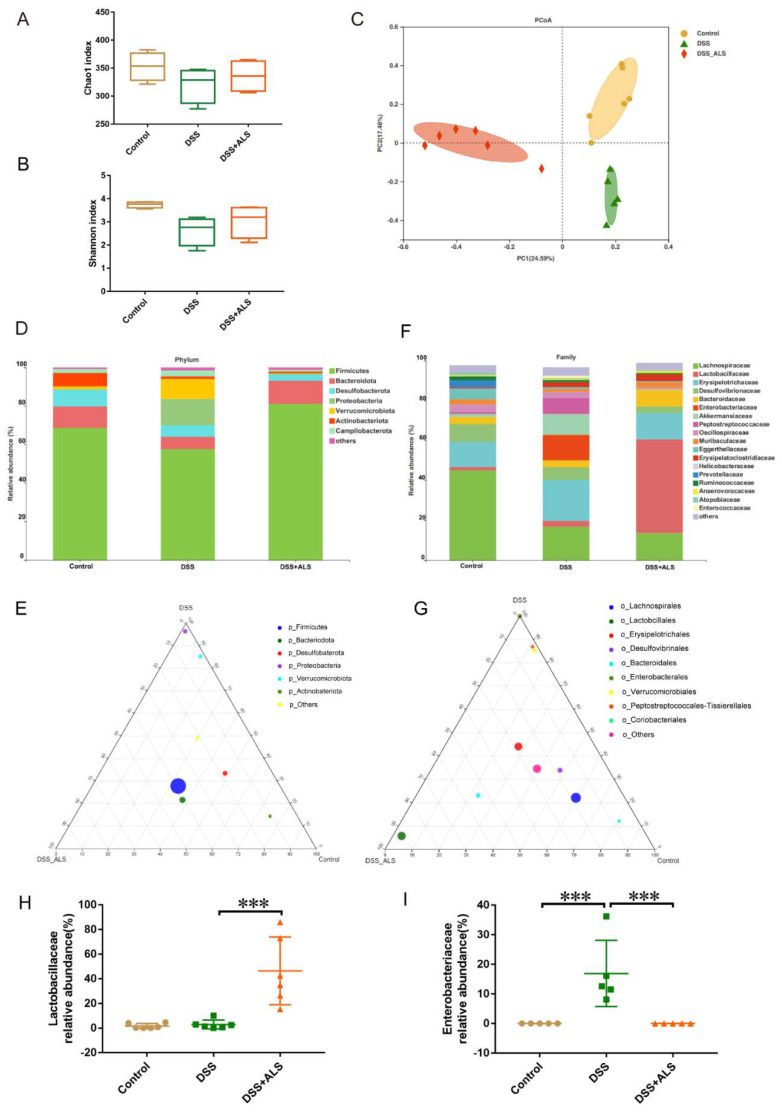
ALS ameliorates gut microbiota dysbiosis in cecum contents. (**A**,**B**) Estimation of gut bacterial richness (Chao1 index) and diversity (Shannon index). (**C**) PCoA plot illustrating gut microbiota composition. (**D**) Phylum level of relative abundance of bacterial species in the cecum content of mice. (**E**) Phylum-level taxonomy illustrated by Ternary plot graph. The relative abundance of the bacteria indicated by the distance between vertex and each circle. (**F**) Family level of relative abundance of bacterial species in the cecum content of mice. (**G**) Family-level taxonomy illustrated by Ternary plot graph. (**H**) Relative abundance of *Lactobacillaceae* in the cecum content. (**I**) Relative abundance of *Enterobateriaceae* in the cecum content. Data are mean ± SEM. *n* = 5–6 mice per group. *** *p* < 0.001.

Emerging evidence indicates the typical association between dysbiosis of gut microbiota and ulcerative colitis [23]. The analysis of the cecum contents by 16S rRNA gene amplicon sequencing showed that ALS treatment did not affect the bacterial richness and diversity (Chao1 and Shanno indices) (Figure 5A,B). However, a significant modulation in bacterium composition was observed in ALS-treated mice, based on the uniFra-based principal coordinates analysis (PCoA) (Figure 5C). The gut bacterial composition at phylum and family levels was further analyzed to better understand the impact of ALS on the microbial community. At the phylum level, ALS treatment significantly reduced the high abundance of *Proteobacteria* induced by DSS (Figure 5D,E). The family-level analysis showed that DSS led to a powerful enrichment of *Enterobateriaceae*, a common signature of the gut microbiota dysfunction, which was remarkably suppressed by ALS treatment (Figure 5F,G,I). Notably, the *Lactobacillaceae*, which belong to the anti-inflammatory bacteria and regulate intestinal regeneration, were the predominant bacterial community at the family level and increased after ALS treatment (Figure 5F–H). These results suggested that ALS supplementation significantly influenced the diversity and composition of the microbiome.

### 3.6. ALS Increases the Production of Microbial Bile Acids (BAs)

**Figure 6:**
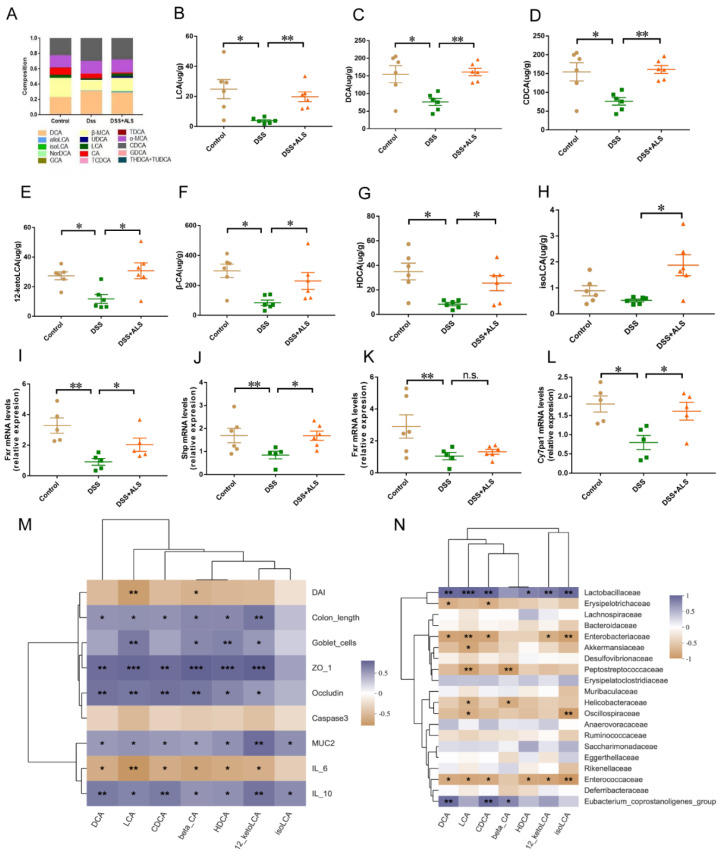
ALS increases the production of microbial Bas. (**A**) The relative abundance of bile acids in the cecum content. (**B**–**H**) Significantly altered BAs quantification (**I**,**J**) Measurement of *Fxr* and *Shp* genes in colon tissues. (**K**,**L**) Measurement of *Fxr* and *Cyp7a1* gene in liver. (**M**) Analysis of the Spearman’ correlation between significantly altered BAs and colitis-related parameters. (**N**) Analysis of Spearman’ correlation between significantly altered BAs and microbiota. Data are mean ± SEM. n = 5–6 per group. * *p* < 0.05, ** *p* < 0.01 and *** *p* < 0.001. n.s., not statistically significant.

The levels of BAs in the stool of mice were subsequently measured using targeted metabolomics. ALS administration showed distinct clustering of the BAs and markedly increased the levels of lithocholic acid (LCA), deoxycholic acid (DCA), chenodeoxycholic acid (CDCA), 12-ketolithocholic acid (12-ketoLCA), β cholic acid (β-CA), hiodeoxycholic acid (HDCA), and isolithocholic acid (isoLCA) compared with the DSS group (Figure 6A–H). Since BAs are farnesoid X receptor (*Fxr*) agonists, which can interact with MAPKs and NF-κB signaling [24,25,26], the impacts of ALS on the activation of FXR and its downstream small heterodimer partner (*Shp*) in the colon was further examined. A significantly upregulated expression of the *FXR* and *Shp* genes was observed in the colon of the ALS-treated group (Figure 6I,J). Additionally, the expression of the cholesterol 7α-hydroxylase (*Cyp7a1*) gene in the liver showed a trend towards an increase in ALS-treated mice, whereas upregulation of the liver *Fxr* gene was observed (Figure 6K,L). FXR is responsible for the amelioration of DSS-induced colitis in mice by restoring gut barrier dysregulation, reducing goblet cell loss, and attenuating colonic inflammation [27]. Our results also showed that BAs were closely related to gut barrier and inflammatory responses (Figure 6M). Thus, Spearman’s correlation analysis was conducted between the markedly increased BAs and the top 20 bacteria to determine the association between fecal BAs and gut dysbiosis. DCA, LCA, CDCA, HDCA, β-CA, 12-ketoLCA, and isoLCA levels were positively correlated with the abundance of *Lactobacillaceae* (Figure 6N), while DCA, LCA, DCA, 12-ketoLCA, and isoLCA levels were inversely correlated with Enterobacteriaceae and *Enterococcaceae* (Figure 6K). The results suggested that ALS treatment resulted in a significant alteration of the fecal BAs closely correlated with the DSS-induced colitis and related intestinal microbiota dysregulation.

### 3.7. Microbiota Ablation with Antibiotics Reduces the Efficacy of ALS Supplementation in Colitis Impairment

**Figure 7:**
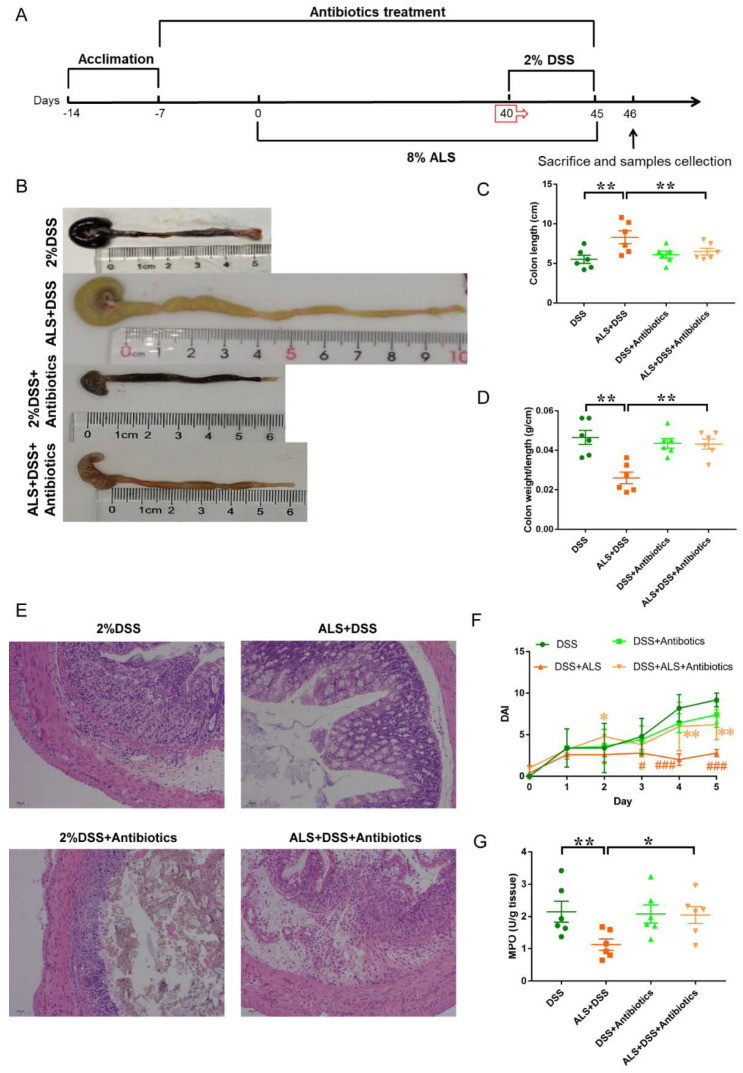
Antibiotics treatment attenuated the protective effects of ALS on DSS-induced colitis. (**A**) Study design of the experiment. (**B**) Pictures of colon samples showing the colon length. (**C**) Colon length. (**D**) Colon weight/length ratio. (**E**) Representative colon histological sections of mice treated with antibiotic or not. (**F**) Disease activity scores. # *p* < 0.05, ### *p* < 0.001, relative to DSS group; * *p* < 0.05, ** *p* < 0.01, relative to DSS+ALS group. (**G**) MPO activity of the colitis mice with antibiotics pretreatment or not. Data are mean ± SEM. *n* = 5–6 mice per group. * *p* < 0.05, and ** *p* < 0.01.

ALS-fed mice were treated with broad-spectrum antibiotics to eliminate the ALS-induced gut microbiota in order to determine whether ALS-induced remission in colitis is associated with an altered microbial community structure (Figure 7A). Antibiotics reduced the efficacy of ALS against DSS-colitis. Antibiotics partially abolished the protective effects of ALS on DSS-induced colon shortening (Figure 7B,C) and the colonic weight/length ratio increase (Figure 7D). Compared with the ALS group, increased colon damage (Figure 7E) and DAI scores (Figure 7F) were observed in mice with colitis from the ALS + Antibiotics group. Additionally, DSS remarkably elevated IBD-associated myeloperoxidase (MPO) activity in the colon (Figure 7G), but ALS administration significantly ameliorated the elevation (Figure 7G). Similarly, antibiotics treatment dampened the alteration in ALS-fed mice (Figure 7G). Collectively, these findings indicated that the gut microbiota was a key mediator in the protection of DSS-induced colitis by ALS, and the microbiome partially contributed to the benefits of ALS. 

## 4. Discussion

IBD is rapidly increasing as a health-threatening disease worldwide. Dietary interventions showed promising results in numerous epidemiological and experimental studies as prominent factors in ulcerative colitis [28,29]. Hence, the development of effective dietary agents and an understanding of the underlying mechanisms is an area of significant interest. This work reports that ALS, as a famous, unique Chinese medicinal and edible fugus, is an appealing candidate for its beneficial effect on intestinal functions. Indeed, ALS promoted an anti-inflammatory state and upregulated the expression of tight-junction-associated proteins and antimicrobial peptides in the colon. The MAPK/NF-κB signaling pathway was involved in the ALS-induced intestinal homeostasis in colitis. Additionally, ALS in the diet regulated the composition of gut microbiota and gut microbiota-related BAs. Broad-spectrum antibiotic treatment reduced the efficacy of ALS-induced protection from colitis. Thus, gut microbiota resulted in being a pivotal mediator in the amelioration by ALS of experimental colitis.

The gut microbiota consists of trillions of microbes with a crucial role in the maintenance of gut homeostasis [30]. Dysregulated intestinal microbiota seems to be implicated in ulcerative colitis [31]. The manipulation of the microbiota may be an effective treatment approach in the management of colitis. Mice were fed a diet supplemented with 8% ALS for 40 days and fecal microbiota was assessed to investigate the impact of ALS in modulating gut microbiota. In line with the findings that microbiota dysbiosis featured an enrichment of harmful bacterial species belonging to Enterobacteriaceae, our data showed that ALS significantly suppressed the increased abundance of *Enterobateriaceae* caused by DSS treatment. The present study also found that ALS improved gut microbiota function, mainly increasing the relative abundance of colonic *Lactobacillaceae*. *Lactobacillaceae* play a critical role in gut homeostasis. Recent studies suggested that supplementation of *Lactobacillaceae* can ameliorate colitis through an increase in mucus thickness, regulation of intestinal regeneration, and abrogation of the pro-inflammatory status [32,33,34], which was also observed in our experiments. Notably, the depletion of gut microbes with antibiotics reduced the efficacy of ALS against DSS-colitis. Antibiotic treatment suppressed the mitigating effects of ALS on the symptoms of colitis, including the shortened colonic length, increased DAI scores, colon damage, and elevated activities of MPO. Antibiotic treatment suggested that gut microbiota is a pivotal mediator in the management of experimental colitis by ALS. Combining the significant effect of ALS on intestinal microbiota and the suppressive effect of antibiotic treatment on the protection of ALS against colitis, we speculated that ALS might ameliorate colitis in a microbiota-dependent manner. 

The gut microbiota is known to influence host physiological functions partially through microbiota-derived molecules, such as BAs, which are greatly impacted by dietary nutrients [35]. Gut microbiota is able to convert endogenous or dietary molecules into metabolites to communicate with the peripheral tissues of the host [36]. BA deficiency induced by dysbiosis in inflammatory-prone IBD patients has been known to promote inflammation in the intestine, which could be converted by BA supplementation [37,38]. BAs can act as signaling molecules to regulate the intestinal function through the activation of BA receptors such as FXR [39]. Thus, focusing attention on gut microbiota-BAs-FXR signaling in the treatment of IBD could be a promising approach. Our results showed that ALS treatment not only led to the enrichment of BAs, such as LCA, DCA, and CDCA, but also the activation of FXR signaling in the colon. Recent work described that the biotransformation of BAs due to gut microbiota can impact the metabolism of BAs in the liver [40]. Our results showed a trend toward increased Cyp7a1 but not FXR. An in-depth analysis of an ALS–gut microbiota–liver/gut BA–metabolism axis would be useful to obtain a comprehensive overview of whether and how changes in the intestinal BA pool induced by ALS can modulate the hepatic synthesis. Activated FXR signaling interacts with MAPKs to inhibit the NF-κB-mediated inflammatory response [41]. The release of endogenous BAs into the gut tract promotes tissue renewal through the promotion of intestinal stem cell renewal [38]. ALS had a potent anti-inflammatory effect in the colon, characterized by a significant decrease in pro-inflammatory cytokines. In addition, the increase in crypt height was found in ALS-treated mice, indicating that ALS supplementation induced enhanced intestinal regeneration after DSS injury. Thus, these data strongly indicated that the interplay between microbiota and ALS to activate the BA–FXR signaling seems to serve as an approach for treating colitis. 

The dysfunction of the intestinal barrier functions can lead to dysregulated responses to gut commensal bacterial and bacterial leakage [42], which are common pathogenic factors of IBDs. Thus, the restoration of intestinal barrier functions is an essential intervention to cure IBD. The effects of ALS on intestinal homeostasis in vivo were also demonstrated in this work. Our data showed that the supplementation of ALS led to an increased expression of tight-junction-associated proteins reducing epithelial permeability. ALS-treated mice displayed restored histological scores with a more-intact crypt structure than the DSS group. Additionally, ALS treatment increased the restoration of goblet cells storing mucin. Goblet cells secret glycosylated Muc2 to form the colonic mucus layer [43]. Mucin produces a coat to cover the intestinal cells, protecting them from the infection of toxic substances and bacteria to maintain intestinal homeostasis [44]. These data therefore strongly support the notion that tight-junction-associated proteins and goblet cell restoration were responsible for ALS intestinal barrier homeostasis. Additionally, our current studies are only limited to the mouse model DSS-induced acute colitis, and ALS should be tested in other advanced preclinical models of IBD in order to represent the clinical manifestations observed in humans in a broader way. Besides, our results only suggest that ALS protects against DSS-induced colitis in a preventive manner and validation in a therapeutic setting is needed. 

## 5. Conclusions

In conclusion, our work revealed that a diet intervention with ALS could protect against DSS-induced colitis by modulating the inflammatory response, barrier restoration, and microbiota dysbiosis. These findings offer initial experimental evidence for the potential use of ALS in intestinal disorders and unveil a new role of ALS in preventing intestinal dysfunctions. 

## Data Availability

The data used to support the findings of this study are included within the article.

## Supplementary Materials

The following are available online at https://www.mdpi.com/article/10.3390/nu13113926/s1, Table S1: The macronutrient composition of the ALS powder in the study, Table S2: The formula of Chow and ALS diets in the study, Table S3: List of primers used in this study.
