# Supplementary material for: Armillaria luteo-virens Sacc Ameliorates Dextran Sulfate Sodium Induced Colitis through Modulation of Gut Microbiota and Microbiota-Related Bile Acids
Source: Nutrients. 2021 Nov 3;13(11):3926. doi: 10.3390/nu13113926 (PMC8623807; doi:10.3390/nu13113926)
Supplement: Supplementary file 1 [file nutrients-13-03926-s001.zip › nutrients-1395854-supplementary.pdf]

Supplementary Materials: The following are available online at https://www.mdpi.com/article/10.3390/nu13113926/s1,Table S1: The macronutrient composition of the ALS powder in the study, Table S2: The formula of Chow and ALS diet in the study Table S3: List of primers used in this study.

Table S1. The macronutrient composition of the ALS powder in the study.

Macronutrient	Unit (g/100g)
Protein	39.2
Fat	8.6
Carbohydrate	41.15
Soluble dietary fiber	26.63
Insoluble dietary fiber	12.65
Moisture	3.85
Ash	7.2

Table S2. The formula of Chow and ALS diet in the study.

Ingredient	Chow diet	ALS diet
Casein	189.58	156.3
L-cystine	2.84	2.84
Corn Starch	298.59	297
Maltodextrin 10	33.18	33.18
Sucrose	331.77	331.77
ALS	0	81
Cellulose	47.4	15
Soybean Oil	23.7	23.7
Lard	18.96	12
Mineral Mix S10026	9.48	9.48
Dicalcium Phosphate	12.32	12.32
Calcium Carbonate	5.21	5.21
Potassium Citrate	15.64	15.64
Vitamin Mix V10001	9.48	9.48
Choline Bitartrate	1.9	1.9
Total	1000	1008.57

[1] Chow and ALS diets are isocaloric and comprised of 20% protein,10% fat and 70% carbohydrate.

Table S3. List of primers used in this study.

Genes	Gene bank accession	Primers	Primers sequence	Annealing temperatures(°C)	Amplicon Size (bp)
Spdef	NM_001357728.1	Forward	5′-CTCGCTAGAGCAGGTGCAAT-3′	54	126
		Reverse	5′-AGCCACTTCTGCACGTTACC-3	54	
FXR	NM_001163504.1	Forward	5′-TCCTTGTCCTCCTCGGAACA-3′	60	166
		Reverse	5′-TGGGGTTTCCTGAAGCCTTG-3′	60	
Cyp7a1	NM_029865.2	Forward	5′-CTGCACCCTGAGAAGCATCC-3′	60	144
		Reverse	5′-CGAGAACTCGCCATACTGGT-3′	59	
Z0-1	XM_021187463.2	Forward	5′-AGTTCTGCCCTCAGCTACCA-3′	54	173
		Reverse	5′-GCTTAAAGCTGGCAGTGTC-3′	51	
Claudin1	NM_016674.4	Forward	5′-CACTTCCAGACTCCACCACC-3′	56	249
		Reverse	5′-CGATCCATCCCAGAGAAGCC-3′	56	
Claudin2	XM_006528490.4	Forward	5′-CTTCGGGACTTCTACTCGCC-3′	56	151
		Reverse	5′-AGTTGGTACGATTGCCCTGG-3′	54	
Claudin4	NM_009903.2	Forward	5′-CGTCATCCGCGACTTCTACA-3′	59	159
		Reverse	5′-GAGTAGGGCTTGTCGTTGCT-3′	60	
Occludin	XM_032898729.1	Forward	5′-ACAAAGAGCTCTCTCGTCTCG-3′	54	192
		Reverse	5′-CATAGTCTCCCACCATCCTC-3′	54	
Klf4	MT592851.1	Forward	5′-AGAACAGCCACCCACACTTG-3′	54	183
		Reverse	5′-CCCTGTGTGTTTGCGGTAGT-3′	54	
Reg3g	NM_011260.2	Forward	5′-CGTGCCTATGGCTCCTATTGCT--3′	57	121
		Reverse	5′-TTCAGCGCCACTGAGCACAGAC-3′	59	
Fut2	NM_001271993.1	Forward	5′-AGTTTCCTTGGTCCTGAACGA-3′	52	131
		Reverse	5′-CTACAGCAATCCTCTTCTGGC-3′	54	
Reg3b	XM_029478855.1	Forward	5′-CCCAGGCTTATGGCTCCTAC-3′	54	191
		Reverse	5′-ATGGAGCCCAATCCAAGTGT-3′	53	
Shp	NM_011850.3	Forward	5′-GCACGATCCTCTTCAACCCA-3′	60	185
		Reverse	5′-CAGAAGGGTGCCTGGAATGT-3′	60	
Defensin 4	NM_010039.2	Forward	5′-AGGCTGATCCTATCCAAAACACA-3′	55	92
		Reverse	5′-TGGCCTCCAAAGGAGATAGACA-3′	53	
